# Bee Bread as a Diuretic

**Published:** 1865-09

**Authors:** James S. Whitmire

**Affiliations:** Metamora, Ill.


ARTICLE XXXI.

### BEE BREAD AS A DIURETIC.

BY JAMES S. WHITMIRE, M.D., Metamora, Ill.

I am not aware that it is generally known to the profession, but it has come very forcibly to my mind, within the two months past, that beebread is a most powerful *diuretic*.

Having bought, from one of my neighbors, a quantity of honey in the comb, for family or table use, we proceeded, accordingly, to feast upon the delicious morsel, every day, for four or five weeks, during which time, on account of the muddy roads, I was compelled to take to the saddle, to perform my professional engagements; and, at the same time, I observed that I could not ride more than four or five miles without being compelled to stop and void my urine. At first, I thought but little of this inconvenience, as equestrian exercise always, to a greater or less extent, tends to promote the secretion of urine, but after a few days of that kind of exercise that effect upon the kidneys would gradually wear away, and the annoyance cease. But this time the abundant secretion continued so persistently that I became alarmed lest my kidneys were becoming diseased, and I began seriously to investigate the nature and cause of my trouble.

I tried, first, the nitric acid test for albumen, supposing I might be affected with albuminuria, but no signs appeared. I then searched for some proof of diabetes mellitus, but without any satisfactory results, until I applied the two delicate tests of the epicure upon it, when, lo, there appeared the taste of beebread upon my tongue, and the flavor of the same upon my olfactories. All this time I was compelled to rise from my bed every night, once or twice, to urinate; and, while complaining to my wife about my troubles and fears in regard to my situation, she informed me in a very laconic manner, as I thought, that she supposed it must be a kind of family disorder, as the children had scarcely missed wetting the bed, every time they slept, for five or six weeks, a thing very uncommon with them for two or three years past.

I now selected some of the oldest comb that contained the greatest quantity of the bread, and separated it from the honey and comb; then, after abstaining a week from the use of my favorite sweet, and getting quite over my renal disease, as well as my unnecessary alarm, I partook of the bread, without the luxury of the honey, to the extent of ʒj, three times per day, when, as I was expecting, back came the enormous secretion, but this time producing an entirely different effect upon my mind, so that I was now prepared to investigate the effects a little more at length. I continued taking ʒiij per day, for about a week, during which time I voided from four to six fluid pounds per day, the difference being the *greatest when I was at some out-door exercise. When I remained quiet*, in my warm office, there was from one to one and a-half pounds less secretion than when exercising. I also repeated the same experiment on my children, and found, to my entire satisfaction, that this article possesses most valuable diuretic powers, and there seemed to be no disagreeable symptoms following its use, excepting a slight degree of flatulency and a looseness of the bowels produced, the latter of which is not, unfrequently, very desirable, particularly in dysuria, where there is irritation of the neck of the bladder and urethra, or, even in strangury, where there is absolute inflammation of the urinary passages. This, to me, is the more evident, from the enormous quantity of urine secreted, and, consequently, any irritating quality that it might contain would be so diluted as to be rendered entirely mild and inoffensive to the delicate structure of the urinary passages.

One advantage this article has over many others of its class is, that it is entirely palatable and inoffensive to the stomach, producing no irritation or nausea of the latter organ, and, consequently, the facility with which it may be administered to children and persons having a delicate stomach.

I write this article, not so much to enlighten the profession, or extend the already long list of diuretic agents, as to introduce a simple and, at the same time, an efficient remedy, that may be found at nearly every farm-house in the land, and to show the novel manner in which my attention was called to its effects.

